# CD4^+^ cell count recovery following initiation of HIV antiretroviral therapy in older childhood and adolescence

**DOI:** 10.1097/QAD.0000000000001905

**Published:** 2018-09-03

**Authors:** Victoria Simms, Sarah Rylance, Tsitsi Bandason, Ethel Dauya, Grace McHugh, Shungu Munyati, Hilda Mujuru, Sarah L. Rowland-Jones, Helen A. Weiss, Rashida A. Ferrand

**Affiliations:** a MRC Tropical Epidemiology Group, London School of Hygiene & Tropical Medicine, London; b Department of International Public Health, Liverpool School of Tropical Medicine, Liverpool, UK; c Biomedical Research and Training Institute, Harare; d Department of Paediatrics, University of Zimbabwe, Harare, Zimbabwe; e Nuffield Department of Medicine, University of Oxford, Oxford; f Clinical Research Department, London School of Hygiene & Tropical Medicine, London, UK.

**Keywords:** adolescent, CD4^+^ cell count, HIV, immune reconstitution

## Abstract

**Objective:**

To investigate CD4^+^ cell count recovery following ART initiation in perinatally HIV-infected children diagnosed in later childhood.

**Design:**

Observational prospective cohort study of newly diagnosed children aged 6–15 in Harare, Zimbabwe.

**Methods:**

Participants were enrolled into a cohort at seven primary healthcare clinics between January 2013 and January 2015. ART was initiated according to national guidelines and CD4^+^ cell counts were performed 6-monthly over 18 months. The relationship between CD4^+^ cell count and time on ART was investigated using regression analysis with fixed (population) and random (individual) effects, and age at ART initiation as a covariate.

**Results:**

Of the 307 participants who initiated ART, the median age at initiation was 11.7 years (interquartile range 9.6–13.8). The addition of an individual intercept and slope as random effects significantly improved the model fit compared with a fixed effects-only model. CD4^+^ response (using a square-root transformation) was best modelled using a two-knot linear spline, with significant effects of time on ART and age at ART initiation. Younger children had a higher CD4^+^ cell count at ART initiation (−17.9 cells/μl per year of age), an accelerated increase during the first 3 months on ART (−38.9 cells/μl per year of age at day 84), and a sustained higher CD4^+^ cell count.

**Conclusion:**

Earlier ART initiation in older children is associated with accelerated CD4^+^ cell count recovery and lasting immune reconstitution. Our findings support WHO guidance recommending ART initiation in all children, irrespective of disease stage and CD4^+^ cell count.

## Abstract 2

Supplemental Digital Content is available in the text

## Introduction

Most children living with HIV acquired the infection perinatally. In the pre-ART era, HIV-positive infants in sub-Saharan Africa had a 50% risk of mortality before age 2 years [1]. However, as HIV epidemics matured, survival estimates were successively revised upwards, and it is estimated that about one-third of perinatally infected infants have ‘slow progressing’ HIV disease, with a median survival of at least 16 years without treatment [2]. Large numbers of children now present with undiagnosed perinatally acquired HIV in older childhood and adolescence in sub-Saharan Africa [3], where over 80% of the world's HIV-infected children live [4].

For infants, ART initiation regardless of CD4^+^ cell count or disease stage substantially reduces mortality [5] and has been recommended since 2010 [6]. In older children and adolescents, the evidence for reduced mortality is less clear. A systematic review of antiretroviral therapy (ART) for children aged under 3 years found few studies [7], and the only trial in older children was underpowered and showed no mortality benefit of early initiation [8]. In the absence of clear evidence, and to simplify HIV treatment programmes, guidance for children aged at least 5 years followed that for adults, with ART initiation recommended for those with clinical WHO stage 3 or 4 disease, or low CD4^+^ cell count (below 350 cells/μl in 2010 [6], with the threshold raised to 500 cells/μl in 2013 [9]).

More recently, the TEMPRANO and START trials showed that ART initiation before CD4^+^ cell count dropped below 500 cells/μl reduced morbidity [10,11] and the HPTN 052 trial showed that it also reduced sexual transmission [12]. As a result, the most recent WHO guidance (from 2016) recommends immediate ART initiation for all individuals living with HIV, irrespective of CD4^+^ cell count and disease stage [13]. Again, the guidance has also been applied to children even though based on evidence from trials conducted in adults.

There are concerns related to long-term toxicity and development of resistance because of poor adherence to ART, particularly for children who will be on ART a decade or more longer than adults initiating ART. Conversely, there are likely benefits of earlier ART initiation. The aim of this study is to estimate change in CD4^+^ cell count over time in a cohort of children diagnosed with HIV in older childhood, and to assess whether age affected CD4^+^ cell count recovery.

## Methods

Participants aged 6–15 years were recruited from seven public sector primary healthcare clinics in Harare, Zimbabwe between January 2013 and January 2015, following provider-initiated HIV testing and counselling. Those who tested HIV-positive were offered enrolment into a prospective cohort study. Exclusion criteria included living outside Harare or choosing not to seek HIV care at one of the study clinics. Participants were invited for follow-up visits at 3-monthly intervals for assessment of clinical status, up until 18 months postenrolment. Within the cohort, participants who consented to home visits were enrolled into the ZENITH randomized controlled trial, reported elsewhere [14].

The guideline in use in January 2013 stated that children should be initiated on ART at a CD4^+^ cell count below 350 cells/μl or if they had WHO stage 3 or 4 HIV disease [6]. In March 2014, Zimbabwe adopted the new WHO guidelines [9], with a revised threshold for ART initiation of 500 CD4^+^cells/μl.

CD4^+^ cell count was measured at 3 and 6 months postinitiation, and otherwise 6-monthly, with an Alere PIMA CD4 (Waltham, Massachusetts, USA) machine. Participant data were recorded by a study nurse on paper clinical report forms and entered into an MS Access database using Cardiff TELEFORM Intelligent Character Optical Mark Recognition Software (Version 10.9). Statistical analyses were performed using R v3.4.1 and Stata v14.0 (StataCorp, College Station, Texas, USA).

To explore the relationship between the CD4^+^ cell count and the explanatory variables, time on ART and age at ART initiation, we performed linear mixed-effects regression modelling of longitudinal data. Time was modelled using a linear spline. Splines provide a flexible way of modelling response curves over time using standard linear mixed modelling software. We used the simplest family of splines, in which the response curve is a continuous, piece-wise linear function. Comparison of nested models was made using the likelihood ratio test. A linear spline model was explored incorporating zero, one or two knots to determine which best fitted the data. The first knot was a priori positioned at time 0, because of the biological plausibility of a change in CD4^+^ response at ART initiation. The optimal position for the second knot was ascertained by calculating the log-likelihood value for a knot on each day.

To build the model, we first used ordinary least squares regression, then added a random intercept and then a random slope. Next we added the spline terms one by one, then age at ART initiation, and finally an interaction between age and time. At each stage, we used a likelihood ratio test to determine whether the increased complexity was justified.

The residuals were plotted against the fitted values for the CD4^+^ response model to verify the assumptions of homogenous variance and normally distributed errors. As a sensitivity analysis, we fitted the same linear spline model to a censored dataset, which consisted only of data between 100 days preinitiation and 600 days postinitiation of ART. This was to ensure that the model was not unduly influenced by sparse data at the extreme ends of the follow-up period.

Ethical approval for the study was obtained from the Medical Research Council of Zimbabwe (MRCZ/A/1676) and the Ethics Committees of Harare City Health Services, the Biomedical Research and Training Institute (AP 108/2012) and the London School of Hygiene and Tropical Medicine (6305).

## Results

Between 23 January 2013 and 23 January 2015, 385 children and adolescents were enrolled into the cohort, of whom 307 (79.7%) initiated ART during follow-up. Reasons for noninitiation were: no CD4^+^ cell count below the threshold for ART initiation (350 cells/μl prior to March 2014, 500 cells/μl thereafter; *n* = 78); less than a month of follow-up before transferring out (*n* = 5) or being lost to follow-up (*n* = 1); only became eligible for ART at the last follow-up visit (*n* = 4).

Participants were aged 6–15 years at enrolment and 6–17 years at ART initiation (Table 1). Of the 307 participants who initiated ART, about half were female patients (52.1%) and almost all (96.7%) acquired HIV perinatally, defined as a history of maternal or natural sibling HIV or death, and self-report of no sexual debut, blood transfusion or surgery [15]. There were a total of 1155 CD4^+^ cell count test results, collected between 898 days prior to ART initiation and 1099 days postinitiation (median 149 days postinitiation, IQR −9 to 343), with a median follow-up per person of 516 days. Most participants (*n* = 206; 67.1%) initiated ART within the first 4 weeks of enrolment. Following Zimbabwean national guidelines, all children aged less than 10 years or weighing less than 25 kg were initiated on zidovudine, all those weighing more than 35 kg were initiated on tenofovir, and those in the middle range were initiated on either (including 10 on d4T).

**Table 1:** Characteristics of study participants (*N* = 307).

Variable	Categories	*N* (%)
*N*		307 (100)
Sex	Male	147 (47.9)
	Female	160 (52.1)
Age at ART initiation (years)	6–9	98 (31.9)
	10–13	146 (47.6)
	14–17	63 (20.5)
CD4^+^ cell count at enrolment (cells/μl)	0–99	49 (16.0)
	100–199	37 (12.1)
	200–349	89 (29.0)
	350–499	66 (21.5)
	500+	66 (21.5)
Time from enrolment to ART initiation	Up to 4 weeks	206 (67.1)
	>4 weeks to 1 year	80 (26.1)
	>1 year	21 (6.8)
ART regimen at initiation	TDF/3TC/EFV	66 (25.3)
	TDF/3TC/NVP	62 (23.8)
	TDF/3TC/ATV	1 (0.4)
	ZDV/3TC/EFV	32 (12.3)
	ZDV/3TC/NVP	90 (34.5)
	d4T/3TC/EFV	3 (1.2)
	d4T/3TC/NVP	7 (2.7)
	Unknown	46
Number of CD4^+^ cell count test results	1	35 (11.4)
	2	30 (9.8)
	3	50 (16.3)
	4	66 (21.5)
	5	111 (36.2)
	6–7	15 (4.9)

ART, antiretroviral therapy.

**Fig. 1:**
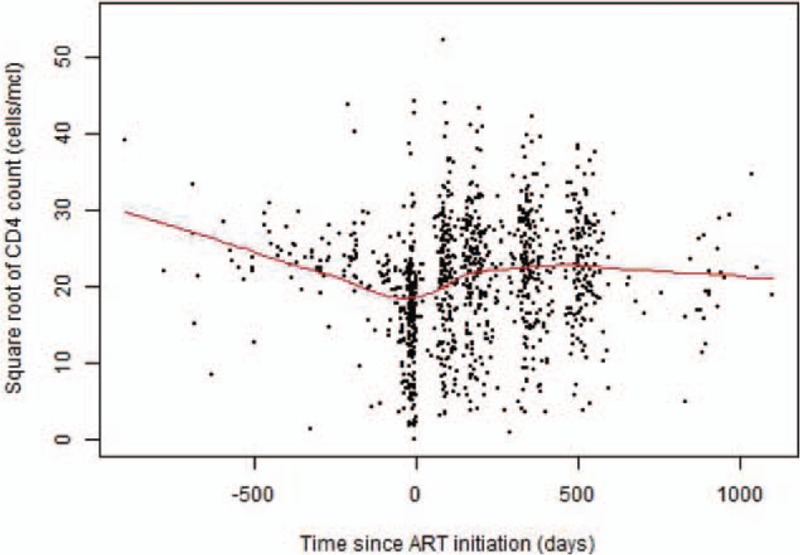
Square root of CD4^+^ cell count over time since antiretroviral therapy initiation with locally weighted scatterplot smoothing (LOWESS) (*N* = 1155 observations).

The square root transformation of CD4^+^ cell count resulted in a more symmetrical data distribution than either the raw values or a log transformation (Fig. 1), and was the selected outcome measure. A random intercept model was a much better fit than a fixed-effects model, with a likelihood ratio test result of 660.1 (*P* < 0.001) A model with a random intercept and slope was slightly better than a random intercept alone (likelihood ratio test = 28.8, *P* < 0.001). The best-fitting linear spline model of CD4^+^ cell count results incorporated two knots, the first fixed a priori at 0 days after ART initiation and the second, established using log-likelihood values, at 84 days postinitiation (Supplementary Figure 1). Compared to a one-knot model the likelihood ratio chi-square value was 198.4 (*P* < 0.001). Age at ART initiation improved the fit both as a main effect (likely-hood ratio test = 24.8, *P* < 0.001) and again as an interaction effect of age at ART initiation with time (likely-hood ratio test = 13.5, *P* = 0.004).

**Fig. 2:**
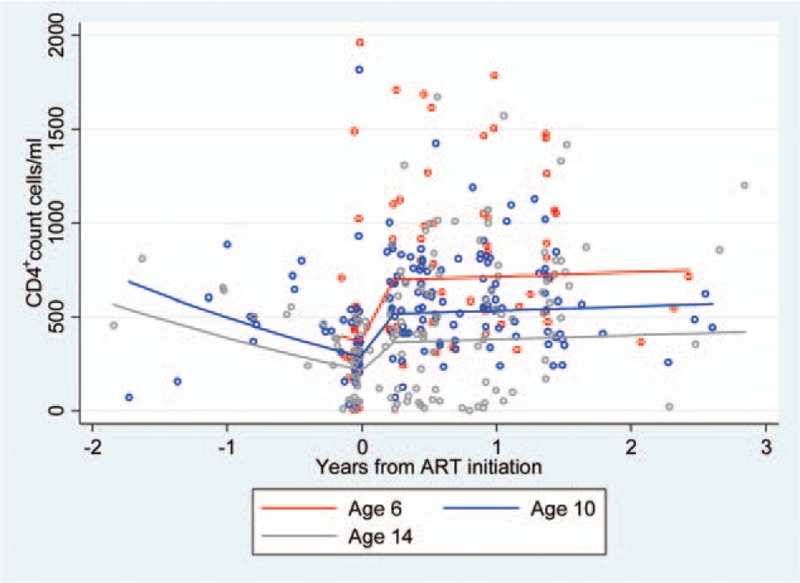
Fitted CD4^+^ cell count over time since antiretroviral therapy initiation, by age at antiretroviral therapy initiation (*N* = 1155 observations).

The final model showed that CD4^+^ cell count decreased over time until ART initiation, then increased until the second knot at day 84, and then remained steady (Table 2). CD4^+^ cell count was significantly associated with age as a fixed effect. On the day of initiation, one additional year of age at ART initiation was associated with a 17.9-point decrease in CD4^+^ cell count. Over time the effect of age on CD4^+^ cell count increased. For example, after 84 days on ART, each one-year increase in age was associated with a decrease in CD4^+^ cell count of 38.9 cells/μl. On day 84 postinitiation, the estimated average CD4^+^ cell count was 696 cells/μl for a child who started ART aged 6 years; 518 cells/μl for a child who started ART aged 10 years; and 332 cells/μl for age at ART initiation of 15 years (Fig. 2).

**Table 2:** Results of linear spline model of square root of CD4^+^ cell count over time around antiretroviral therapy initiation (*N* = 1155 observations).

Parameter	Value (95% CI)	SE	*P* value
Days pre-ART	−0.02 (−0.04 to 0.00)	0.01	0.08
0–84 days post-ART	0.13 (0.09–0.17)	0.02	<0.01
>84 days post-ART	−0.11 (−0.15 to −0.08)	0.02	<0.1
Age at ART initiation (years)	−0.54 (−0.84 to −0.25)	0.15	<0.01
Interaction of days pre-ART with age at ART initiation	0.00 (−0.00 to 0.00)	0.00	0.74
Interaction of 0–84 days post-ART with age at ART initiation	−0.01 (−0.01 to −0.00)	0.00	0.01
Interaction of >84 days post-ART with age at ART initiation	0.00 (0.00–0.01)	0.00	0.01
Intercept	22.38 (18.97–25.79)	1.74	<0.01

ART, antiretroviral therapy; CI, confidence interval.

Before ART initiation, the interaction effect of age at initiation was statistically significant but negligible in its effect. Once participants were on ART the trajectories diverged, with younger children gaining a greater rise in CD4^+^ cell count (a difference in square root of CD4^+^ of 0.005, *P* = 0.01).

For the sensitivity analysis, 120 outlier observations at the extreme ends of the observation period were dropped, and the models were refitted (Supplementary Table 1). Results were similar (Supplementary Figure 2). Residuals of both models were unbiased and homoscedastic.

## Discussion

Among children and adolescents living with HIV in Zimbabwe, CD4^+^ cell count increased in the first 3 months following ART initiation, and then stabilized. The first 3 months of ART appear to be a key window for immune reconstitution in children. Immune reconstitution in children is qualitatively and mechanistically different from adults, probably because of greater involvement of the naïve T-cell pool and a more active thymus gland [16]. T cells are formed in bone marrow and migrate to the thymus where they mature. From childhood, the thymus begins to atrophy, although it still produces some new T cells throughout life. Adults have a biphasic response to ART, with a rapid increase in memory CD4^+^ cells followed by a slower, smaller increase in naïve CD4^+^ cells [17]. In contrast, children demonstrate peripheral CD4^+^ repopulation through increased thymic naïve T-cell output, with only a small increase in memory CD4^+^ cells [17,18]. For this reason, the reference curves for CD4^+^ cell count response in the first 9 months of ART, based on data from Europe [18], cannot be applied to children.

Children who initiated ART at a younger age showed a greater immediate benefit, which was sustained over time. Among adults, older age has also been associated with poorer CD4^+^ response to ART [18], but not to the extent observed among children in this study. In our study, older participants had lower CD4^+^ cell counts at ART initiation, and poorer immunological recovery in the critical 3-month period after initiation. As a result, the difference in CD4^+^ cell count between age groups was greater in participants who were stable on ART than among those not yet initiated on ART. The missed opportunity for recovery in older children and adolescents had long-lasting consequences, as they remained at a lower average CD4^+^ cell count to the end of follow-up.

For this cohort, low CD4^+^ cell count at baseline was a risk factor for other adverse outcomes, including increased risk of hospital admission and mortality, as shown previously [19]. Of 13 children who died during follow-up, 11 had a CD4^+^ cell count below 350 cells/μl at enrolment [20]. There are two possible causes of the lower CD4^+^ cell count at baseline and poorer immune recovery of adolescents compared with younger children. Firstly, among perinatally infected children, older age corresponds to later diagnosis. Children who were older at ART initiation had, therefore, been immunosuppressed for a longer period. Secondly, adolescents may have limited capacity for rapid immunological recovery, as thymic involution has already advanced but a memory T-cell reserve is not yet established. HIV-infected children have a smaller thymus than HIV-exposed but uninfected children at age 3 months [21], and early thymic involution is associated with rapid disease progression [22]. Delayed ART initiation in children may thus have a dual effect. As well as prolonging the duration of untreated infection, it also raises the age of ART initiation, and so limits the child's capacity for immunological recovery. This would explain why a year's delay in a child has more effect on CD4^+^ cell count than a year's delay for an adult.

Our results are comparable with other studies exploring CD4^+^ cell count reconstitution following ART initiation in children. Recent studies of HIV-infected children starting ART in Uganda, Zimbabwe [23], and Europe [24] have described a nonlinear model for CD4^+^ reconstitution, with a rapid rise in CD4^+^ cell count reaching a plateau, which varied by age. However, a quarter of children did not demonstrate this asymptotic immune reconstitution. In these studies, the investigators fitted a nonlinear mixed-effects model, which predicted higher long-term CD4^+^ cell counts for children starting ART at a younger age and with a higher initial count [23,24], and the trajectory of increase was steeper in younger children. This is consistent with our finding that age at ART initiation was associated with both the level of CD4^+^ cell count at ART initiation and the rate of change in CD4^+^ cell count after ART initiation. The cohort from Uganda and Zimbabwe, part of the ARROW trial, benefited from a longer follow-up (median 4 years) with CD4^+^ cell count measured every 12 weeks [23].

Strengths of this study include the prospective cohort design, and the fact that participants received care in the public sector, making the findings more generalizable. The use of splines revealed that CD4^+^ recovery is concentrated within a short 3-month period, and the use of interaction and random slopes allowed for the effect of age on CD4^+^ cell count to change over time. Longer term follow-up of cohorts are needed to measure the effects of ART toxicity or resistance development, and to understand the long-term CD4^+^ response as children grow up. Failure to achieve long-term CD4^+^ cell count reconstitution is associated with poor clinical outcomes. Perinatally infected cohorts in the USA had a higher rate of advanced disease-defining events and mortality at lower CD4^+^ cell counts, up to age 30 [25]. Even small CD4^+^ cell count deficits are associated with increased risk of cardiovascular disease, stroke, and cancer in adult HIV patients [26,27]. Additionally, CD4^+^ cell count is only one element of immunological health. A study of children and adolescents in Kenya found that after ART initiation, CD4^+^ cell count quickly recovered to levels normal for the HIV-negative population, but CD4^+^:CD8^+^ ratio and plasma-soluble CD14^+^ did not [28].

A recent review of the benefits and risks of universal ART for children concluded that there is evidence of reduced morbidity, improvement of growth, and better immune recovery [29]. However, risks remain in terms of drug toxicity, development of resistance because of poor adherence, and the challenges of implementation, especially in resource-limited settings. This study adds to the evidence on immunological decline and reconstitution in children, and provides clinical evidence to support the policy of universal ART in older children and adolescents to maximise the potential gains from treatment.

## Acknowledgements

We thank all participants and caregivers.

Funding: This work was supported by the Wellcome Trust (Grant number 095878/Z/11/Z).

### Conflicts of interest

There are no conflicts of interest.

## Supplementary Material

Supplemental Digital Content

## References

[1] Newell M-L Coovadia H Cortina-Borja M Rollins N Gaillard P Dabis F Mortality of infected and uninfected infants born to HIV-infected mothers in Africa: a pooled analysis. *Lancet* 2004; 364:1236–1243. 15464184 10.1016/S0140-6736(04)17140-7

[2] Ferrand RA Corbett EL Wood R Hargrove J Ndhlovu CE Cowan FM AIDS among older children and adolescents in Southern Africa: projecting the time course and magnitude of the epidemic. *AIDS* 2009; 23:2039–2046. 19684508 10.1097/QAD.0b013e32833016ce PMC3408596

[3] Ferrand RA Munaiwa L Matsekete J Bandason T Nathoo K Ndhlovu CE Undiagnosed HIV infection among adolescents seeking primary healthcare in Zimbabwe. *Clin Infect Dis* 2010; 51:844–851. 20804412 10.1086/656361 PMC4578231

[4] UNAIDS. AIDS by the numbers. Joint United Nations Programme on HIV/AIDS. Geneva: UNAIDS; 2016.

[5] Violari A Cotton MF Gibb DM Babiker AG Steyn J Madhi SA CHER Study Team. Early antiretroviral therapy and mortality among HIV-infected infants. *N Engl J Med* 2008; 359:2233–2244. 19020325 10.1056/NEJMoa0800971 PMC2950021

[6] World Health Organization. Antiretroviral therapy for HIV infection in infants and children: towards universal access. 2010 revision. Geneva: World Health Organization; 2010. 23741772

[7] Penazzato M Prendergast AJ Muhe LM Tindyebwa D Abrams E Optimisation of antiretroviral therapy in HIV-infected children under 3 years of age. *Cochrane Database Syst Rev* 2014; 5:CD004772. 24852077 10.1002/14651858.CD004772.pub4 PMC11022182

[8] Puthanakit T Saphonn V Ananworanich J Kosalaraksa P Hansudewechakul R Vibol U PREDICT Study Group. Early versus deferred antiretroviral therapy for children older than 1 year infected with HIV (PREDICT): a multicentre, randomised, open-label trial. *Lancet Infect Dis* 2012; 12:933–941. 23059199 10.1016/S1473-3099(12)70242-6 PMC3541427

[9] World Health Organization. Consolidated guidelines on the use of antiretroviral drugs for treating and preventing HIV infection. Geneva: World Health Organization; 2013. 24716260

[10] Danel C Moh R Gabillard D Badje A Le Carrou J Temprano ANRS Study Group. A trial of early antiretrovirals and isoniazid preventive therapy in Africa. *N Engl J Med* 2015; 373:808–822. 26193126 10.1056/NEJMoa1507198

[11] Lundgren JD Babiker AG Gordin F Emery S Grund B INSIGHT START Study Group. Initiation of antiretroviral therapy in early asymptomatic HIV infection. *N Engl J Med* 2015; 373:795–807. 26192873 10.1056/NEJMoa1506816 PMC4569751

[12] Cohen MS Chen YQ McCauley M Gamble T Hosseinipour MC Kumarasamy N HPTN 052 Study Team. Prevention of HIV-1 infection with early antiretroviral therapy. *N Engl J Med* 2011; 365:493–505. 21767103 10.1056/NEJMoa1105243 PMC3200068

[13] World Health Organization. Consolidated guidelines on the use of antiretroviral drugs for treating and preventing HIV infection. 2nd ed. Geneva: World Health Organization; 2016. 27466667

[14] Ferrand RA Simms V Dauya E Bandason T McHugh G Mujuru H Community-based caregiver support to reduce the risk of virological failure among children and adolescents with HIV infection in Harare, Zimbabwe (ZENITH): a randomised controlled trial. *Lancet Child Adol Health* 2017; 1:175–183. 10.1016/S2352-4642(17)30051-2 PMC5656092 29104904

[15] McHugh G Rylance J Mujuru H Nathoo K Chonzi P Dauya E Chronic morbidity among older children and adolescents at diagnosis of HIV infection. *J Acquir Immun Defic Syndr* 2016; 73:275–281. 10.1097/QAI.0000000000001073 PMC5065928 27171738

[16] De Rossi A Walker AS Klein N De Forni D King D Gibb DM Increased thymic output after initiation of antiretroviral therapy in human immunodeficiency virus type 1-infected children in the Paediatric European Network for Treatment of AIDS (PENTA) 5 Trial. *J Infect Dis* 2002; 186:312–320. 12134227 10.1086/341657

[17] Douek DC McFarland RD Keiser PH Gage EA Massey JM Haynes BF Changes in thymic function with age and during the treatment of HIV infection. *Nature* 1998; 396:690–695. 9872319 10.1038/25374

[18] Bouteloup V Sabin C Mocroft A Gras L Pantazis N Le Moing V Standard Reference Distribution of CD4 Response to HAART Project Team for the Collaboration of Observational HIV Epidemiological Research Europe (COHERE) in EuroCoord. Reference curves for CD4 T-cell count response to combination antiretroviral therapy in HIV-1-infected treatment-naive patients. *HIV Med* 2017; 18:33–44. 27625009 10.1111/hiv.12389

[19] McHugh G Simms V Dauya E Bandason T Chonzi P Metaxa D Clinical outcomes in children and adolescents initiating antiretroviral therapy in decentralised healthcare settings in Zimbabwe. *J Int AIDS Soc* 2017; 20:21843. 28872269 10.7448/IAS.20.1.21843 PMC5719665

[20] Walker AS Gibb DM Monitoring of highly active antiretroviral therapy in HIV infection. *Curr Opin Infect Dis* 2011; 24:27–33. 21150591 10.1097/QCO.0b013e3283423e0e

[21] Meyers A Shah A Cleveland RH Cranley WR Wood B Sunkle S Thymic size on chest radiograph and rapid disease progression in human imunodeficiency virus 1-infected children. *Pediatr Infect Dis* 2001; 20:1112–1118. 10.1097/00006454-200112000-00004 11740315

[22] Kourtis AP Ibegbu C Nahmias AJ Lee FK Clark WS Sawyer MK Early progression of disease in HIV-infected infants with thymus dysfunction. *N Engl J Med* 1996; 335:1431–1436. 8875920 10.1056/NEJM199611073351904

[23] Picat M-Q Lewis J Musiime V Prendergast A Nathoo K Kekitiinwa A ARROW Trial Team. Predicting patterns of long-term CD4 reconstitution in HIV-infected children starting antiretroviral therapy in sub-Saharan Africa: a cohort-based modelling study. *PLoS Med* 2013; 10:e1001542. 24204216 10.1371/journal.pmed.1001542 PMC3812080

[24] Lewis J Walker AS Castro H De Rossi A Gibb DM Giaquinto C Age and CD4 count at initiation of antiretroviral therapy in HIV-infected children: effects on long-term T-cell reconstitution. *J Infect Dis* 2012; 205:548–556. 22205102 10.1093/infdis/jir787

[25] Neilan AM Karalius B Patel K Van Dyke RB Abzug MJ Agwu AL Pediatric HIV/AIDS Cohort Study and the International Maternal Adolescent and Pediatric AIDS Clinical Trials Network. Association of risk of viremia, immunosuppression, serious clinical events, and mortality with increasing age in perinatally Human Immunodeficiency Virus-infected youth. *JAMA Pediatr* 2017; 171:450–460. 28346597 10.1001/jamapediatrics.2017.0141 PMC5411314

[26] Lichtenstein KA Armon C Buchacz K Chmiel JS Buckner K Tedaldi EM HIV Outpatient Study (HOPS) Investigators. Low CD4+ T cell count is a risk factor for cardiovascular disease events in the HIV outpatient study. *Clin Infect Dis* 2010; 51:435–447. 20597691 10.1086/655144

[27] Guiguet M Boue F Cadranel J Lang J-M Rosenthal E Costagliola D Clinical Epidemiology Group of the FHDH-ANRS CO4 cohort. Effect of immunodeficiency, HIV viral load, and antiretroviral therapy on the risk of individual malignancies (FHDH-ANRS CO4): a prospective cohort study. *Lancet Oncol* 2009; 10:1152–1159. 19818686 10.1016/S1470-2045(09)70282-7

[28] Alvarez P Mwamzuka M Marshed F Kravietz A Ilmet T Ahmed A Immune activation despite preserved CD4 T cells in perinatally HIV-infected children and adolescents. *PLoS One* 2017; 12:e0190332. 29287090 10.1371/journal.pone.0190332 PMC5747457

[29] Barlow-Mosha L Musiime V Davies M-A Prendergast AJ Musoke P Siberry G Universal antiretroviral therapy for HIV-infected children: a review of the benefits and risks to consider during implementation. *J Int AIDS Soc* 2017; 20:21522. 10.7448/IAS.20.1.21552 PMC5527851 28691434

