# Supplementary material for: CD4+ cell count recovery following initiation of HIV antiretroviral therapy in older childhood and adolescence
Source: AIDS. 2018 Sep 3;32(14):1977–82. doi: 10.1097/QAD.0000000000001905 (PMC6125740; doi:10.1097/QAD.0000000000001905)
Supplement: Supplemental Digital Content [file aids-32-1977-s001.doc]

**Supplementary Figure 1: Log likelihood values to determine the optimal position of the second knot in the CD4 cell response linear spline model**


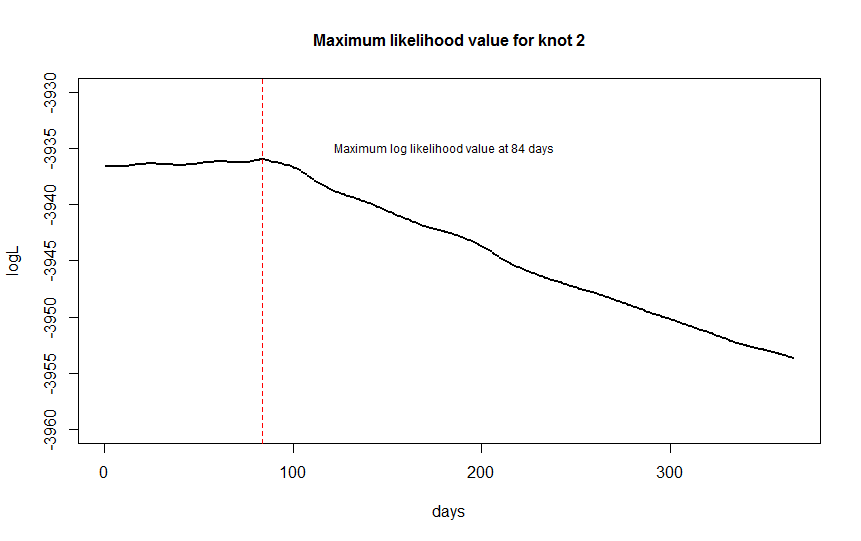


**Supplementary Figure 2: Fitted CD4 count over time since ART initiation in dataset with outliers removed, by age at ART initiation (N=1035 observations)**

## Supplementary Table 1: results of linear spline model of square root of CD4 count over time around ART initiation, with outliers removed (N=1035 observations)

| **Parameter** | **Value (95% CI)** | **SE** | **p-value** |
| --- | --- | --- | --- |
| **Days from ART initiation** | -0.01 (-0.11, 0.10) | 0.05 | 0.87 |
| **Spline 1** | 0.12 (-0.01, 0.26) | 0.07 | 0.07 |
| **Spline 2** | -0.12 (-0.16, -0.07) | 0.02 | <0.01 |
| **Age at ART initiation (years)** | -0.58 (-0.93, -0.23) | 0.18 | 0.01 |
| **Days from ART initiation*age at ART initiation** | -0.00 (-0.01, 0.01) | 0.01 | 0.77 |
| **Spline 1*age at ART initiation** | -0.00 (-0.01, 0.01) | 0.01 | 0.61 |
| **Spline 2*age at ART initiation** | 0.01 (0.00, 0.01) | 0.00 | 0.02 |
| **Intercept** | 22.55 (18.51, 26.59) | 2.06 | <0.01 |
